# Interesting to Parents

**Published:** 1881-11

**Authors:** 


### INTERESTING TO PARENTS.

Of the boys who were applicants for enlistment as apprentices in the United States Navy, during the year 1879, twenty per thousand were rejected, on account of venereal diseases, twelve being syphilitics.

